# Role of genetic modifiers in Lafora progressive myoclonus epilepsy-a neurodegenerative disorder with defects in carbohydrate metabolism

**DOI:** 10.1186/1753-6561-6-S3-P43

**Published:** 2012-06-01

**Authors:** Shweta Singh, Subramaniam Ganesh

**Affiliations:** 1 Department of Human Biology, International Medical University, Bukit Jalil, 57000, Kuala Lumpur, Malaysia; 2 Department of Biological Sciences and Bioengineering, Indian Institute of Technology, Kanpur, India

## 

Lafora progressive myoclconus epilepsy, also known as Lafora disease (LD), is the most severe and fatal form of progressive myoclonus epilepsy with its typical onset during the late childhood or early adolescence. LD is characterised by the presence of abnormal glycogen inclusions – called the polyglucosan bodies – in the neurons and various other affected tissues. Therefore defects in the glycogen metabolism have been suspected to underlie neuropathology in LD. The two genes known for LD have been extensively characterized; these are the *EPM2A* and *NHLRC1* genes, coding for a protein phosphatase (named laforin) and an E3 ubiquitin ligase (named malin) respectively. Laforin and malin interact with each other and as a complex participate in diverse cellular pathways. The pathways include glycogen metabolism, endoplasmic reticulum (ER) stress, ubiquitin-proteasome pathways and in heat shock response. A number of disease causing mutations, more than 100 of them, are known in *EPM2A* and *NHLRC1*and several studies have looked at possible genotype-phenotype correlations in the LD families. The factors other than the defective LD gene could possibly be involved in modifying the disease onset or the progression in some cases. Indeed, a recent study demonstrates that a sequence variant in the *PPP1R3C* gene coding for the protein targeting to glycogen (PTG) contributes to the milder course of LD. It would be therefore important to check possible contributions of sequence variations in critical regulators of these pathways which interact with laforin and/or malin and test their possible role as modifiers in LD. Here we have extensively reviewed the literature and list potential modifier genes for LD, their cellular functions, and propose their possible effect in LD. Validating these hypothesis will help us in identifying “druggable targets” for delaying the onset and/or the severity of LD if not its treatment.

